# Impact of intensive lifestyle intervention on gut microbiota composition in type 2 diabetes: a *post-hoc* analysis of a randomized clinical trial

**DOI:** 10.1080/19490976.2021.2005407

**Published:** 2021-12-29

**Authors:** Shaodong Wei, Asker Daniel Brejnrod, Urvish Trivedi, Martin Steen Mortensen, Mette Yun Johansen, Kristian Karstoft, Allan Arthur Vaag, Mathias Ried-Larsen, Søren Johannes Sørensen

**Affiliations:** a Section of Microbiology, Department of Biology, University of Copenhagen, Copenhagen, Denmark; b National Food Institute, Technical University of Denmark, Kgs Lyngby, Denmark; c Skaggs School of Pharmacy and Pharmaceutical Sciences, La Jolla, CA, USA; d Center of Inflammation and Metabolism and Center for Physical Activity Research, Copenhagen University Hospital - Rigshospitalet, Copenhagen, Denmark; e Department of Clinical Pharmacology, Bispebjerg Hospital,University of Copenhagen, Copenhagen, Denmark; f Steno Diabetes Center Copenhagen, Gentofte, Denmark

**Keywords:** exercise, gut microbiota, lifestyle intervention, metformin, physical activity, standard care, type 2 diabetes

## Abstract

Type 2 diabetes (T2D) management is based on combined pharmacological and lifestyle intervention approaches. While their clinical benefits are well studied, less is known about their effects on the gut microbiota. We aimed to investigate if an intensive lifestyle intervention combined with conventional standard care leads to a different gut microbiota composition compared to standard care alone treatment in individuals with T2D, and if gut microbiota is associated with the clinical benefits of the treatments. Ninety-eight individuals with T2D were randomized to either an intensive lifestyle intervention combined with standard care group (N = 64), or standard care alone group (N = 34) for 12 months. All individuals received standardized, blinded, target-driven medical therapy, and individual counseling. The lifestyle intervention group moreover received intensified physical training and dietary plans. Clinical characteristics and fecal samples were collected at baseline, 3-, 6-, 9-, and 12-month follow-up. The gut microbiota was profiled with 16S rRNA gene amplicon sequencing. There were no statistical differences in the change of gut microbiota composition between treatments after 12 months, except minor and transient differences at month 3. The shift in gut microbiota alpha diversity at all time windows did not correlate with the change in clinical characteristics, and the gut microbiota did not mediate the treatment effect on clinical characteristics. The clinical benefits of intensive lifestyle and/or pharmacological interventions in T2D are unlikely to be explained by, or causally related to, changes in the gut microbiota composition.

## Introduction

The prevalence of type 2 diabetes (T2D) has increased rapidly over the past decades.^1^ Successful management of T2D improves individuals’ life expectancy and quality.^2^ The basic principles of T2D management are based upon a combination of pharmacological treatments and lifestyle interventions.^3^ The medical approach includes the preferred first-line medication metformin and potentially second-line medications, *e.g*. glucagon-like peptide-1 (GLP-1) analogue, to manage blood glucose levels. Healthy lifestyle involves increased physical activity levels and dietary changes aiming at a substantial weight loss. Many studies have reported that intensive lifestyle interventions can reduce the incidence of T2D,^4–6^ prevent further deterioration of impaired glucose tolerance,^5,7^ decrease the hemoglobin A1c (HbA1c) level,^8^ and reduce the medication use in overt T2D.^9^

Accumulating evidence suggests that the gut microbiota have specific signatures for individuals with T2D,^10^ hence it raises the question if these two treatments (pharmacological treatments or intensive lifestyle interventions) have different impacts on the gut microbiota. For example, the first-line glucose-lowering medication (GLMED) metformin was shown to significantly affect gut microbiota, *e.g*. increase of *Escherichia* spp., *Akkermansia muciniphila*, and decrease of *Intestinibacter* spp.,^10–12^ and it has been suggested that the therapeutic benefits might be partly mediated *via* the gut microbiota.^13^ Similarly, lifestyle interventions with a healthy diet and/or physical exercise training have also been shown to impact the gut microbiota composition. Diet, in particular, is a key determinant of the gut microbiota composition as dietary patterns (western or vegetarian diet), specific foods (fruits, grain, or vegetables), and food constituents (fiber, protein, or fat) can all lead to changes in the gut microbiota.^14^ Increased exercise levels are associated with enhanced fuel mobilization, muscle glucose uptake, and fat oxidation,^15^ and affect gastric emptying through interleukin-6 (IL-6), leading to reduced postprandial glycemia.^16^ However, it is still unclear how exercise influences gut microbiota.^17^ Professional athletes with extreme exercise (*e.g*. rugby training athletes) and associated diets have higher gut microbial diversity, with enhanced production of amino acids and short-chain fatty acids (SCFAs) (likely contributed by genus *Roseburia* and family XIII Incertae Sedis), compared to more sedentary individuals.^18,19^ Recently, a study has linked extreme exercise (marathon running) with increased gut microbe *Veillonella atypica*,^20^ although further validation is needed.^21^ However, only few randomized clinical trials have evaluated the effects of regular exercise on the microbiome in human populations, especially in individuals with T2D, and in these studies, the effects of exercise have been limited.^22–24^

We previously showed that an intensive lifestyle intervention in individuals with T2D maintained glycemic control while reducing the need for GLMED, and improved clinical characteristics compared to the standard care.^9^ In this *post hoc* analysis, we aimed to investigate how an intensive lifestyle intervention with a subsequent reduction in GLMED impacts the gut microbiota composition compared to standard care in individuals with T2D, and whether gut microbiota composition changes may contribute to the observed clinical treatment benefits.

## Results

### Taxonomic composition differences between treatments

At baseline 0 month (M0), the gut microbiota compositions of individuals in the two treatment groups were not significantly different and no taxa differed in their relative abundances between groups at any taxonomic levels (from phylum to amplicon sequence variant [ASV], adjusted *P* (*P*_adj_) > .05). Specifically, at the phylum level, Firmicutes was the most abundant phylum (71.2% [lifestyle, median] *vs*. 74.3% [standard care, median], *P_adj_* = .921), followed by Actinobacteria (5.6% *vs*. 5.5%, *P_adj_* = .909), Proteobacteria (2.6% *vs*. 2.6%, *P_adj_* = .842), Bacteroidetes (2.6% *vs*. 0.7%, *P_adj_* = .755), and Verrucomicrobia (1.0% *vs*. 0.6%, *P_adj_* = .839). These five phyla accounted for the majority of the gut microbiota abundances (98.9% [lifestyle] and 99.2% [standard care]).

**Figure 1.**
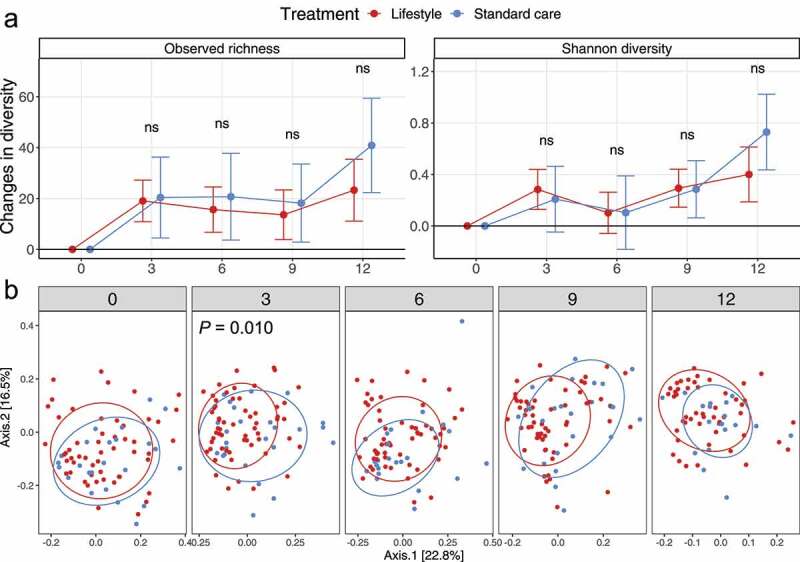
**Taxonomic composition differences between treatments over time (0, 3, 6, 9, 12 months)**. (a) Change of alpha diversity for each individual averaged within groups compared to baseline (M0). The dots refer to the mean and error bars refer to the 95% confidence interval. For the sake of interpretation, if the error bar overlaps the horizontal line (value of zero), it indicates that the change of alpha diversity is not significantly different from zero (no change). The comparison alpha diversity between a time point and baseline was performed with paired two-sided t-test. The comparison of changes in alpha diversity between treatments was performed with two-sided t-test and the significance was shown as “ns” (*P* > .05). (b) Distribution of samples based on weighted UniFrac distance visualized with principal coordinates analysis (PCoA) with ellipses indicating 75% confidence regions for clusters. The *P* value is from the multivariate permutational analysis of variance (PERMANOVA)

To investigate if the two treatments shifted gut microbiota similarly, we compared the changes in observed richness and Shannon diversity between groups at all time windows (Figure 1a, from baseline to 3 months [M0-M3], to 6 months [M0-M6], to 9 months [M0-M9], and to 12 months [M0-M12]). However, none of these changes were significantly different between groups (*P* > .05, shown with “ns”). Next, we assessed the changes in alpha diversity for each treatment. From M0 to M12, richness increased 17.1% in the lifestyle group (105.0 ± 30.3 [M0] *vs*. 123.0 ± 38.7 [M12], mean ± SD; 17.1% = [(123.0–105.0)/105.0] × 100) and 42.9% in standard care (91.8 ± 33.3 [M0] *vs*. 131.2 ± 30.3 [M12]; 42.9% = [(131.2–91.8)/91.8] × 100). Specifically, both treatments had increased richness at M3 compared to M0 (lifestyle, 105.0 ± 30.3 [M0] *vs*. 122.1 ± 30.4 [M3], mean ± SD, *P* < .001; standard care, 91.8 ± 33.3 [M0] *vs*. 110.8 ± 31.2 [M3], *P* = .014), then slightly reduced until M9 (119.7 ± 34.9 [lifestyle], *P* = .007; 107.3 ± 34.5 [standard care], *P* = .022), thereafter increased quickly from M9 to M12. We also investigated ASVs (shown at the genus level) that were responsible for the increased richness (Figure S1) and found that they were mainly from the genera *Bacteroides, Ruminococcaceae UCG 014, Alistipes*, and *Roseburia*. From M0 to M12, Shannon diversity increased 11.3% in the lifestyle intervention (3.28 ± 0.51 [M0] *vs*. 3.65 ± 0.61 [M12], mean ± SD; 11.3% = [(3.65–3.28)/3.28] × 100) and 23.5% in standard care (3.06 ± 0.53 [M0] *vs*. 3.78 ± 0.50 [M12]; 23.5% = [(3.78–3.06)/3.06] × 100). Only individuals in the lifestyle group showed increased Shannon diversity at M3 compared to M0 (3.28 ± 0.51 [M0] *vs*. 3.55 ± 0.47 [M3], mean ± SD, *P* < .001) and standard care did not induce significant difference during the first three months (3.06 ± 0.53 [M0] *vs*. 3.28 ± 0.70 [M3], *P* = .106). Both treatments increased Shannon diversity significantly at M9 (119.7 ± 34.9 [lifestyle], *P* < .001; 3.33 ± 0.63 [standard care], *P* = .014) and M12.

Additionally, we assessed the gut microbiota composition based on the weighted UniFrac distance, visualized by principal coordinates analysis (PCoA), stratified by time point (Figure 1b). Two treatments did not lead to different gut microbiota compositions at M12 (*P* = .127) and other time points (*P* = .138 [M6], *P* = .063 [M9]), except for M3 (*P =* .010). We also compared the distance for each patient at different time windows as a way to assess the changed beta diversity over time (Figure S2). However, none of the changed beta diversities were significantly different between groups at all time windows (*P* > .05).

We then compared the group variance based on weighted UniFrac distance, by assessing the change of group dispersion (mean UniFrac distance to group centroid in multivariate space) over time (Figure S3). When comparing the differences between treatments in the change of sample dispersion, no significant difference was observed at M12 or any other time points (*P* > .05, shown with “ns”, in all conditions). From M0 to M12, both treatments led to reduced group dispersion (0.276 ± 0.103 [M12] *vs*. 0.316 ± 0.085 [M0], *P* = .022, lifestyle, [mean ± SD]; 0.232 ± 0.058 [M12] *vs*. 0.316 ± 0.085 [M0], *P* = .001, standard care). Specifically, for the lifestyle intervention, the group dispersion was significantly reduced at M3 (0.271 ± 0.068, *P* = .003), M9 (0.270 ± 0.091, *P* = .006), and M12 (0.276 ± 0.103, *P* = .022), compared to M0 (0.316 ± 0.085). For standard care, no significant change was observed at M3 (0.300 ± 0.072, *P* = .532), M6 (0.308 ± 0.109, *P* = .743), or M9 (0.311 ± 0.084, *P* = .743), compared to M0 (0.316 ± 0.085).

Next, we found that due to the large group dispersion, the inter-individual variation at M0 was higher than the intra-individual development between M0 and M12 (Figure S4, 0.442 ± 0.159 [M0] *vs*. 0.404 ± 0.206 [M0-M12], *P* = .019, lifestyle, median ± IQR [interquartile range]; 0.460 ± 0.185 [M0] *vs*. 0.320 ± 0.201 [M0-M12], *P* = .010, standard care, median ± IQR), or M0 and M3 (0.442 ± 0.159 [M0] *vs*. 0.302 ± 0.189 [M0-M3], *P* < .001, lifestyle; 0.460 ± 0.185 [M0] *vs*. 0.352 ± 0.205 [M0-M3], *P* = .001, standard care), based on the weighted UniFrac distance.

Finally, we compared the change of taxa abundances between treatments at different taxonomic levels (from phylum to ASV) and different time windows (M0-M3, M0-M6, M0-M9, and M0-12). However, no taxa differed in the changes of their relative abundances at any time windows (*P_adj_* > .05, in all conditions).

### Functional composition differences between treatments

Based on the phylogenetic investigation of communities by reconstructing unobserved states (PICRUSt, version 2) program,^25^ we assessed the imputed functional composition of microbial communities. A total of 428 enzyme-catalyzed reactions (ECs) and 147 KEGG orthologs (KOs) were selected and their changes of relative abundances in time windows between treatments were compared, with the same method as the taxa abundances comparison. It showed that no ECs and KOs had significantly different changes in their relative abundances at any time windows (*P_adj_* > .05, in all conditions). In addition, we also grouped enzyme-catalyzed reactions into pathways and the changes of relative abundances for 193 pathways were compared. Again, no pathways had significantly different changes in their abundances at any time windows (*P_adj_* > .05, in all conditions).

### Clinical characteristics and differences between treatments

In contrast to the similar changes in gut microbiota taxonomic and functional compositions between treatments, clinical characteristics, as reported earlier, changed differently during the 12-month intervention (M0 *vs*. M12).^9,26–28^ Specifically, individuals randomized to lifestyle intervention decreased their HbA1c levels by 3.4 ± 7.5 mmol/mol (mean ± SD) and reduced their need for and use of pharmacological treatments (GLMED score decreased by 1.6 ± 1.6). Intensive lifestyle interventions were in addition associated with increased physical fitness by 6.5 ± 5.7 ml/kg/min, and improved body composition by increasing lean mass (0.7 ± 2.4 kg) and decreasing fat mass (6.3 ± 6.5 kg) (Table S1). In contrast, individuals randomized to standard care either did not progress toward improved health status (*e.g*. GLMED score increased 0.5 ± 1.9), or to a less extent (*e.g*. physical fitness increased 0.3 ± 4.9 ml/kg/min, fat mass decreased 1.5 ± 4.7 kg).

To further describe the changing trend of clinical characteristics, we assessed the change of characteristics over time compared to M0 within each individual and grouped by treatments (Figure S5). We found that the change in clinical characteristics largely occurred during the first three months for individuals in the lifestyle intervention group. In contrast, the characteristics of individuals allocated to the standard care were quite stable during the whole intervention period. Additionally, we also compared the difference in the change of clinical characteristics between treatments, and most of them started being different between treatments from M3 and remained until M12 (Figure S5).

### Gut microbiota composition was shifted in both treatments

**Figure 2.**
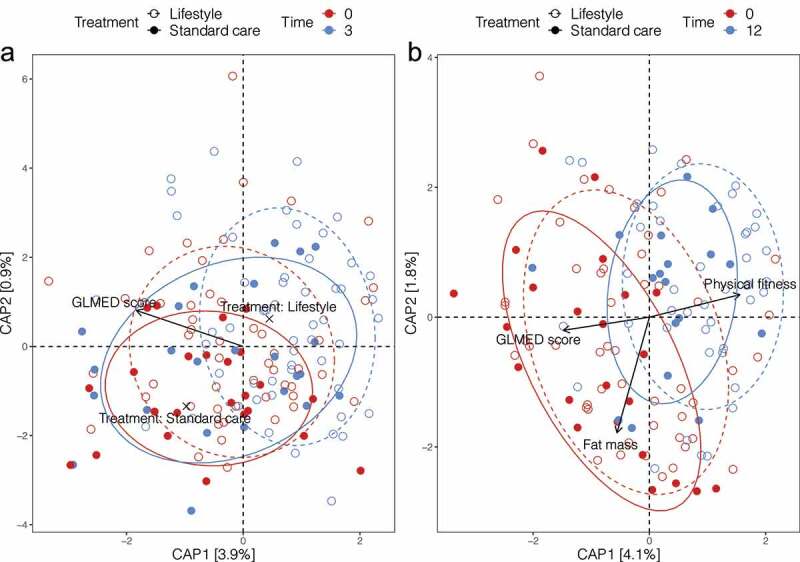
**The weighted UniFrac distance-based redundancy analysis (dbRDA) showing the distribution of samples at M0 and M3** (a) **or M0 and M12** (b)**, and the associations with clinical characteristics and/or treatments**. Each dot is a sample. Colors refer to different time points. Solid dots and solid lines refer to lifestyle intervention; hollow dots and dashed lines refer to standard care. Ellipses indicate 75% confidence regions for clusters. The direction and magnitude of arrows indicate which clinical characteristics that taxa abundance responds to the most strongly. The black crosses in (a) indicate the direction and magnitude of treatments

Next, we performed a distance-based redundancy analysis (dbRDA) to assess the change of gut microbiota composition from M0 to M3 or M12, and interrogated if such changes could be correlated with clinical characteristics and/or treatments (Figure 2). Of all assessed characteristics (Figure S5, Table S2), only GLMED score significantly explained a proportion of variance at M3. Besides, the diabetes treatments (lifestyle intervention and standard care) were also significantly correlated (Figure 2a). While, at M12, significantly correlated factors were GLMED score, physical fitness, and fat mass (Figure 2b). In agreement with the arrows’ direction (characteristics increasing gradient), individuals from the lifestyle intervention significantly reduced the GLMED score, fat mass, and significantly increased physical fitness over time (Figure S5). Individuals from the standard care also tended to change in the beneficial direction (*e.g*. increased physical fitness, decreased fat mass), though none of these characteristics changed significantly over time.

Samples from both treatments largely overlapped with each other both at M0 (R^2^ = 0.010, *P* = .613) and M12 (R^2^ = 0.022, *P* = .130), except M3 (R^2^ = 0.032, *P* = .010, also shown in Figure 1b). However, they all differed significantly from their baseline at both M3 (R^2^ = 0.040, *P* < .001, M0 *vs*. M3, lifestyle; R^2^ = 0.047, *P* = .026, M0 *vs*. M3, standard care) and M12 (R^2^ = 0.074, *P* < .001, M0 *vs*. M12, lifestyle; R^2^ = 0.105, *P* < .001, M0 *vs*. M12, standard care). To investigate which taxa were responsible for the changed beta diversity at M3 and M12 in comparison with M0, we compared the relative abundances between time points for each treatment (Table S3). We observed that many genera changed their abundances significantly at M3 or M12, such as *Bacteroides, Blautia, Roseburia, Escherichia*, and *Faecalibacterium*. Besides, we also observed a sharp reduction of Firmicutes to Bacteroidetes ratio for both treatments at M3 and M12 (26.9 ± 62.2 [M0, median ± IQR], 8.0 ± 10.4 [M3], 4.7 ± 6.1 [M12], *P* < .001 for both M3 and M12 against M0, lifestyle; 63.9 ± 92.1 [M0], 8.1 ± 15.2 [M3, *P* = .003], 7.8 ± 5.5 [M12, *P* < .001], standard care).

### Gut microbiota alpha diversity does not correlate with clinical characteristics

**Figure 3.**
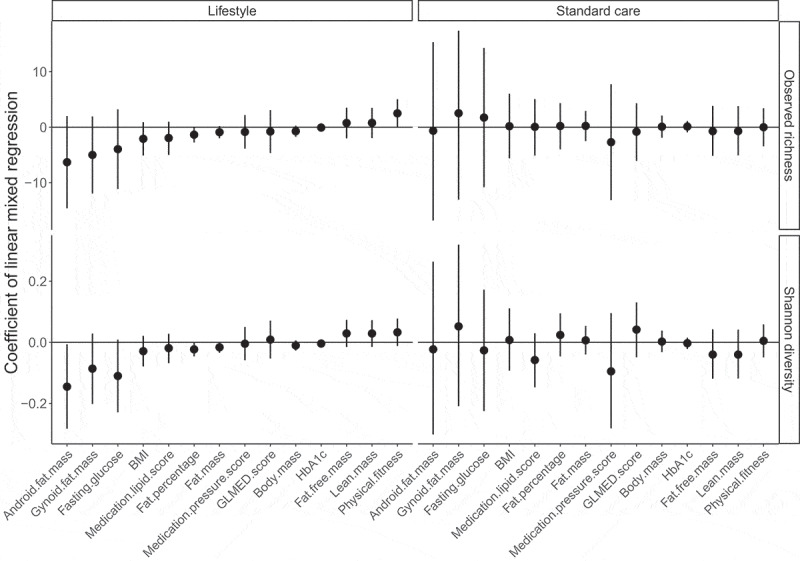
**The correlations between the change of clinical characteristics and the change of alpha diversity**. The Y axis is the correlation coefficient obtained from the linear mixed models, the effects of age, gender, and time windows were adjusted, and patient was used as the random effect. X axis is the clinical characteristic. Vertical lines are the 95% confidence interval of the correlation coefficient mean. A correlation is significant if its confidence interval does not overlap the black horizontal line (value of zero referring to no correlation)

The gut microbiota alpha diversity has been suggested as an indicator of health status.^29,30^ To validate if this notion is reflected in our study, we assessed the correlations between the shift in gut microbiota alpha diversity and the change in clinical characteristics for each treatment (pooling all time windows together), which is shown in Figure 3. Overall, almost none of these correlations was significant (*P* > .05, Table S4). In detail, body fat-related characteristics, such as android fat mass, gynoid fat mass, fat percentage, and fat mass tended to be inversely correlated with gut microbiota alpha diversity in the lifestyle group; The lean mass, fat-free mass, and physical fitness positively correlated with alpha diversity in the lifestyle group. However, these correlations were almost inverted in the standard care group. HbA1c showed quite small correlation coefficients in both groups and were far from being significant (Table S4). GLMED score showed negative correlations with observed richness and positive correlations with Shannon diversity in both groups, but none of them were significant.

### Mediation analysis of gut microbiota

Our study showed clear relevant changes in a number of clinical characteristics (Figure S5). However, it remains unknown the extent to which gut microbiota may mediate any of these clinical benefits and/or differences under the treatment effects. We, therefore, applied a robust distance-based mediation analysis to evaluate the overall mediation effect of gut microbiota. Among all mediation conditions (see Materials and methods), only one mediation came out as statistically significant, namely the treatments’ effect on GLMED score at M3, which appeared to be significantly mediated by the gut microbiota at this specific time point M3 (*P* = .030). However, this specific effect was not further validated by another mediation method “SparseMCMM” (*P* = .505, overall mediation effect).

### Gut microbiota differences stratified by GLMED score change

Based on our dbRDA (Figure 2) and mediation analysis, the clinical characteristic GLMED score possibly best reflected gut microbiota change related to treatments. When considering a reduction of GLMED score at M12 as an indicator of a successful lifestyle change, 17.6% of individuals treated with standard care (three out of 17 persons reduced GLMED score) changed their lifestyle without supervision. Similarly, 23.3% of individuals in the lifestyle intervention (10 out of 43 persons did not reduce GLMED score) failed to change lifestyles. Therefore, to assess the actual impact of lifestyle interventions on gut microbiota, we re-grouped individuals into GLMED score groups: Same (N = 8), Increase (N = 16), and Decrease (N = 36) based on the change of GLMED score (individuals not having samples at both M0 and M12 were removed) (Figure S6). To assess if the direct effect of GLMEDs confounded our GLMED score grouping, we assessed the influence of different intensities of mediation treatment on the gut microbiota by comparing the alpha diversity between different medication intensities (GLMED score 1 to 6) and medication discontinuation (GLMED score 0) (Figure S7, Table S2). In most of the conditions, GLMED did not lead to different gut microbiota alpha diversity compared to GLMED free individuals (*P* > .05).

By comparing the GLMED groups (decrease *vs*. increase), we observed that the changing trend of gut microbiota Shannon diversity only differed at M3 (Figure S8a), the change of group dispersion differed at M3 and M9 (Figure S8b), gut microbiota beta diversity differed at M3 (*P* = .002, Figure S8c), the change of relative abundance of KOs, ECs, and metabolic pathways did not differ at any time windows. Among all assessed taxonomic levels, only very few taxa changed their relative abundances differently between groups (Table S5).

## Discussion

After the 12-month trial, we did not observe statistical differences in the change of gut microbiota composition between the intensive lifestyle intervention and standard care. The gut microbiota alpha diversity did not correlate with the clinical characteristics, and the gut microbiota did not mediate the treatment effects on the clinical characteristics.

### Two treatments led to similar gut microbiota changes

After 12 months of intervention, the two groups did not differ in their gut microbiota in any relevant aspects. (i) The change of alpha diversity was not different between groups at M12 (Figure 1a). (ii) The beta diversity was not different between groups at M12 (Figure 1b). (iii) The change of group dispersion was not different between groups at M12 (Figure S3). (iv) The change of taxa abundance at all assessed taxonomic levels was not different between groups at M12. (v) The change of functional abundance was not different between groups at M12. (vi) Based on our GLMED score grouping, potentially a way to better reflect actual lifestyles, almost none of the above-mentioned outcome measurements differed between GLMED score groups (increase *vs*. decrease) at M12 (Figure S8).

However, we did observe some weak differences in the gut microbiota at M3 between treatments, such as the changing trend of Shannon diversity compared to baseline (Figure 1a), beta diversity between groups (Figure 1b), change of group dispersion compared to baseline (Figure S3), and the dbRDA analysis (Figure 2a). Other than gut microbial changes during the first three months, the shift of clinical characteristics also largely occurred during the first three months (Figure S5). Individuals allocated to lifestyle interventions almost improved all these clinical characteristics during this time window compared to standard care. Thereafter, the improvement in clinical characteristics remained stable and barely further improved.

Importantly these time-wise co-occurred changes of gut microbiota and clinical characteristics at M3 do not necessarily imply that gut microbiota and clinical characteristics were associated, since we almost did not observe any significant associations between the change of the gut microbiota alpha diversity and the change of clinical characteristics, and many of these associations were not consistent between treatments (positive or negative) (Figure 3). Indeed, the lack of differences in gut microbiota composition between treatments after 12 months makes any causal effects of gut microbiota on clinical outcomes highly unlikely.

### Do the two treatments beneficially alter gut microbiota?

After a 12-month intervention, both treatments were associated with extensive and potentially beneficial gut microbiota changes compared to the baseline pre-treatment gut microbiota profiles. The changes included increased richness (17.1% [lifestyle] and 42.9% [standard care]) and Shannon diversity (11.3% [lifestyle] and 23.5% [standard care]). Generally, increased gut microbial diversity and richness have been considered metabolically beneficial,^29,30^ which in turn could have contributed to the beneficial clinical outcomes for both treatments after 12 months. In support of this, the dbRDA analysis revealed that along with the change of gut microbiota composition over time, the physical fitness was improved and the fat mass and GLMED score were reduced (Figure 2b).

A number of taxa were significantly shifted over time (Table S3), which likely reflected the clinical benefits of treatments. For example, *Bacteroides* has been suggested to be beneficial for glucose metabolism in mice,^31,32^ and negatively associated with T2D in humans.^33–35^ In our study, both lifestyle intervention and standard care led to increased occurrence and abundance of *Bacteroides* (Figure S1, Table S3). *Roseburia* was reported to be higher in abundances in healthy control than diabetic individuals.^10,36^ In the present study, *Roseburia* was the top genus contributing to the increased richness and was more abundant at M3 and M12 than baseline (Figure S1, Table S3). Other genera were possibly also involved in the improvement of clinical outcomes, such as *Blautia, Escherichia*, and *Faecalibacterium*.^37,38^ To be noted, though two diabetes treatments (lifestyle intervention and standard care) indeed changed the gut microbiota composition along with potential beneficial effects on clinical outcomes, there are few data, if any, to suggest that the clinical benefits are caused directly by the changes in the gut microbiota composition.^39^

Besides, although controversial,^37,40^ the ratio between Firmicutes and Bacteroidetes has been positively associated with obesity and T2D in previous studies.^41–45^ In this study, the Firmicutes to Bacteroidetes ratio in both treatments decreased dramatically in the supposed beneficial direction.

### Treatments likely do not impact clinical characteristics through gut microbiota

Although the GLMED score was observed to be significantly mediated by the gut microbiota at M3 based on the distance-based mediation analysis, such mediation effect was not confirmed using an alternative method SparseMCMM (*P* = .505). Therefore, we do not have sufficient statistical evidence to prove a mediation effect. Even if we consider GLMED score as being mediated by the gut microbiota, the mediation effect of gut microbiota does not seem to be strong, considering that 14 clinical characteristics (shown in Figure S5) were assessed, but only GLMED score was significant. A better application of mediation analysis would be to assess the mediation effect between the change of gut microbiota and the change of clinical characteristics; however, to our knowledge, such a longitudinal mediation method is not yet available.^46^

### Difficulties in inferring general principles

In our study, it seems that general and strong associations were not present between gut microbiota and clinical characteristics or treatments. A few reasons may have contributed: (i) We observed that the differences between individuals at baseline M0 were larger than the differences within individuals between M0 and M3, or between M0 and M12, based on weighted UniFrac distance (Figure S4). It is consistent with the study by Taniguchi et al.^24^ that the effect of exercise was not greater than the individual variation. Thus, high inter-individual variation may lead to reduced detection power. But our paired t-test, paired Wilcoxon rank sum test, and linear mixed model have avoided or taken into account such inter-individual variation. A good control of influencing factors (*e.g*. medications, diet) for gut microbiome would help decrease the large individual variation. (ii) Small effect size of physical exercise on the gut microbiota makes it difficult to detect differences between treatments. Prior studies only reported “subtle”^23^ and “modest”^22^ effect of exercise on the gut microbiota. Increasing the sample population and the intensity of lifestyle interventions would facilitate the detection of microbial differences. (iii) Lack of adherence to treatments, derived from that *e.g*. not all patients in lifestyle intervention group changed their lifestyles and not all patients in standard care group continued their lifestyles, decreased the ability to differentiate changed lifestyles from constant lifestyles. However, this is alleviated by regrouping individuals based on the GLMED score change and consistent results were observed as compared to the intention-to-treat therapy-based grouping (Figure S8). A direct measurement of lifestyles for both treatments, such as daily calories intake, footsteps, and physical activity intensities, would help regroup and compare patients based on the actual lifestyles. (iv) The use of the 16S rRNA gene sequencing technique has limited us to estimate the microbial functions (though can be predicted with PICRUSt2, but still with limitations, https://github.com/picrust/picrust2/wiki/Key-Limitations) and does not provide enough resolution (*e.g*. strain level) to detect differences. Including other “omics” techniques would certainly increase the assessment dimensions.

## Conclusions

In this *post-hoc* analysis, we did not observe significant differences in the change of gut microbiota composition between intensive lifestyle and standard care interventions after 12 months, except for a weak difference observed at the three-month follow-up. Gut microbiota did not correlate with assessed clinical characteristics and did not appear to mediate the treatment benefits on clinical characteristics. Overall, our results imply that clinical benefits of intensive lifestyle interventions and/or conventional standard care with multifactorial and pharmacological treatments, were unlikely to be attributable to the observed changes in the gut microbiota.

## Materials and methods

### Study design, participants, and randomization

This is a *post-hoc* analysis of a randomized clinical trial^9,26–28^ where the primary aim was to investigate the effect of an intensive lifestyle intervention on glycemic control. A total of 878 individuals were screened for inclusion (Figure S9). Based on the inclusion criteria (*e.g*. above 18 years old, T2D diagnosis for less than 10 years) and exclusion criteria (*e.g*. HbA1c level greater than 9%, insulin-dependence), 98 participants were included in the study. Participants were randomized to either standard care alone or intensive lifestyle and standard care in a ratio of 1:2, respectively (stratified by sex). To reduce the confounding influence of dysregulated baseline HbA1c levels, GLMED was standardized to obtain glycemic control at least six weeks prior to baseline assessment. A full protocol paper has been published.^47^ This study was performed in Region Zealand and the Capital Region of Denmark from April 2015 to August 2016. This study was approved by the Scientific Ethical Committee at the Capital Region of Denmark (clinicaltrials.gov registration: NCT02417012). All participants provided oral and written informed consent.

### Outcomes for the *post-hoc* microbiome analysis

The outcomes were: (i) Differences in gut microbiota taxonomic and functional compositions (or changes in compositions) between treatments at each time point (or time window). (ii) Gut microbiota changes from baseline to 12 months for each lifestyle and standard care treatment. (iii) Correlation and mediation analysis of gut microbiota with the treatments and/or the clinical characteristics.

### Interventions

The intensive lifestyle intervention has been previously described in detail elsewhere.^47^ Briefly, all participants received standard care, such as education in T2D and blinded treat-to-target medical regulation based on a pre-specified algorithm. Participants from both groups were encouraged to be physically active in their leisure time (≥ 10,000 steps per day).

In addition, the intensive lifestyle group participants additionally completed five to six weekly aerobic sessions whereof two to three sessions were combined with resistance training. All exercise sessions were supervised during the first four months, and supervision was gradually reduced throughout the intervention period. Also, participants in the lifestyle intervention group were given a hypocaloric diet with a macronutrient distribution of 45% to 60% carbohydrate, 15% to 20% protein, and 20% to 35% fat (< 7% saturated fat). During the first 4 months the total energy intake was restricted (−500 kcal/day relative to individually calculated energy need) and thereafter an isocaloric diet was given, aiming for a body mass index of 25 kg/m^2^. Participants were also asked to register their diet and weigh their food. Individual and group-based dietary counseling were offered by clinical dietitians and progressively reduced over time. Food frequency questionnaire was used for both groups at baseline and 12 months. Participants in the standard care group were not monitored for their daily diet or physical activity.

### Assessment of outcomes and fecal samples

The gut microbiota was assessed by 16S rRNA gene sequencing of the fecal samples collected at baseline (M0), 3- (M3), 6- (M6), 9- (M9), and 12-month (M12) follow-up (Figure S9). Basic techniques and principles used to study the microbiota in a clinical context were previously reviewed.^48^ The clinical characteristics were collected at the same time point as the fecal samples and the baseline clinical characteristics are shown in Table S6.

The fecal samples were taken by the participants themselves at home with OMNIgene·GUT|OM-200 kit and immediately stored in the freezer at −20°C. The samples were transported (30–45 min) in insulated envelopes alongside a freezer pack and brought to the lab by participants along with the visit. The feces collection date was as close to participants’ scheduled visits as possible and not earlier than 48 hours. The samples were immediately stored at −80°C upon arrival in the lab.

### Sample population

In total, 413 fecal samples were collected from 98 individuals, of which 389 were successfully sequenced (19,884 ± 9,180 sequences [mean ± SD], samples lower than 2,000 sequences were removed, Figure S10),^49^ from 86 individuals (age [54.3 ± 8.9, years], mean ± SD; sex male 52.7%; N = 60 [lifestyle] *vs*. N = 26 [standard care]), having at least three samples among the five time points (M0, M3, M6, M9, and M12) (Figure S9). Baseline characteristics of the included individuals by groups are shown in Table S6.

### DNA extraction, sequencing, and bioinformatics

The total microbial DNA was extracted using the PowerMag® Soil DNA Isolation Kit on the EpMotion® 5075vt automated pipetting system (Eppendorf). The V3-V4 region of 16S rRNA gene was amplified by a two-step PCR procedure (30 + 15 cycles) using the modified broad range primers Uni341F (5ʹ-CCTAYGGGRBGCASCAG-3ʹ) and Uni806R (5ʹ-GGACTACHVGGGTWTCTAAT-3ʹ).^50,51^ The PCRBIO HiFi polymerase was used for PCR with the recommended reaction setup and cycling conditions. The amplified products were purified with Agencourt AMPure XP Beads (Beckman Coulter Genomics, MA, USA), normalized with SequalPrep™ Normalization Plate (96) Kit (Invitrogen), and pooled in sequencing libraries with up to 192 samples, including positive (mock communities, Figure S11a) and negative (blank DNA extraction and blank PCR, Figure S11b) controls. The concentration of the pooled libraries was then determined using the Quant-iT™ High-Sensitivity DNA Assay Kit (Life Technologies). Paired-end sequencing was performed on the Illumina MiSeq System (Illumina Inc., Ca, USA) with 5.0% PhiX. All reagents used were from the MiSeq Reagent Kits v2 (Illumina Inc., CA, USA). Adaptors and sequencing primers of raw FASTQ files were removed using “cutadapt” (version 1.15).^52^ Trimmed reads were denoised and assembled into amplicon sequence variants (ASVs) using a modified QIIME2 (version 2018.2) implementation of the DADA2 pipeline, where 8 nucleotides were removed at the 5ʹ end of both forward and reverse, without 3ʹ truncation, the default overlap length of forward and reverse reads was decreased to six nucleotides, and all other parameters were set as default.^53,54^ Taxonomy was assigned against the Silva database (SSU Ref NR 99, release 132).^55^

## Statistical analysis

Alpha diversity (observed richness and Shannon diversity, at the ASV level) was assessed with R-package “phyloseq” after sample size rarefaction (function “rarefy_even_depth”),^56^ the change of alpha diversity compared to baseline within each treatment was tested with paired t-test, and the comparison of changes between treatments was performed with t-test. Their associations with clinical characteristics were performed with a linear mixed effect model with R-package “lmerTest” (age, gender, and time point were included as covariates; patient was included as the random effect).^57^ For beta diversity (at the ASV level), weighted UniFrac distance was calculated with function “diversity beta-phylogenetic” in QIIME2,^53^ tested with permutational multivariate analysis of variance (PERMANOVA) with “adonis” (R-package “vegan”).^58–60^ The dispersion of samples was assessed with the function “betadisper” (R-package “vegan”). The change of dispersion within treatments and comparison of changes between treatments was tested with the same method as for alpha diversity. The comparison of individual variation and individual development based on UniFrac distance was performed with Wilcoxon rank sum test (R-package “stats”). The functional potential of the gut microbiota was predicted with phylogenetic investigation of communities by reconstruction of unobserved states (PICRUSt2) and identified as enzyme-catalyzed reaction (EC), KEGG ortholog (KO), and metabolic pathways (MetaCyc).^25,61–63^ ECs, KOs, and metabolic pathways were removed before further analysis when their relative abundances were lower than 0.1%. The comparison change in functional composition and metabolic pathways between groups was performed with Wilcoxon rank sum test. The weighted UniFrac distance-based redundancy analysis (dbRDA) was performed with the function “ordinate” in R-package “phyloseq” to correlate gut microbiota with clinical characteristics. The clinical characteristics that significantly explain proportions of variance in the constrained ordination were selected with the function “ordistep” in R-package “vegan”. The change of clinical characteristics within treatments and the comparison of changes between treatments was tested in the same way as for alpha diversity. The mediation analysis was performed with R-package “MedTest”,^64^ to assess if treatment effect on the outcome is mediated by the mediator. Specifically, the mediation analysis was performed at each time point with standard care and lifestyle intervention as the treatments, the value of each clinical characteristic as the outcome (assessed characteristics are shown in Figure S5), and the weighted UniFrac distance as the mediator gut microbiota. Besides, we also permutated the mediator and outcome at different time points, which means that the mediator gut microbiota at a time point was tested against clinical characteristics at all time points (*e.g*. gut microbiota at M3 can be tested against GLMED score at M12) to account for possible delayed mediation effect. The significant results derived from the MedTest were validated with another mediation method “SparseMCMM” (R-package SparseMCMM),^65^ done at the genus level and only for genera having higher than 0.1% of relative abundances, the *P* value was obtained with 100 times permutations. The comparison of taxa abundances or Firmicutes to Bacteroidetes ratio between time points within treatments was performed with paired Wilcoxon rank sum test. The comparison of alpha diversity across the glucose-lowering mediation (GLMED) score was tested with paired Wilcoxon rank sum test. The Benjamini-Hochberg correction to control false discovery rate for multiple testing^66^ was only applied for taxonomic, functional potential and metabolic pathway analysis (shown as *P_adj_*). The significance level was set at 0.05 (two-sided statistical tests). The ASVs whose prevalence lower than 1% (present in less than 4 samples) were not included in all the analyses. Most plots were generated with R-package “ggplot2”.^67^

## Supplementary Material

Supplemental Material

## Data Availability

Sequencing data are available from Sequence Read Archive under bioproject ID PRJNA751485 (https://www.ncbi.nlm.nih.gov/sra/PRJNA751485). All individual-level data from cohort participants are protected under Danish and European law and cannot be made openly available. However, relevant data can be made available to other researchers under a data transfer agreement as a collaboration effort.
