# Supplementary material for: Impact of intensive lifestyle intervention on gut microbiota composition in type 2 diabetes: a post-hoc analysis of a randomized clinical trial
Source: Gut Microbes. 2021 Dec 29;14(1):2005407. doi: 10.1080/19490976.2021.2005407 (PMC8726663; doi:10.1080/19490976.2021.2005407)
Supplement: Supplemental Material [file KGMI_A_2005407_SM3849.docx]

**Supplementary materials:**

**Title:** Impact of intensive lifestyle intervention on gut microbiota composition in type 2 diabetes: A *post-hoc* analysis of a randomized clinical trial

**Authors**: Shaodong Wei, Asker Daniel Brejnrod, Urvish Trivedi, Martin Steen Mortensen, Mette Yun Johansen, Kristian Karstoft, Allan Arthur Vaag, Mathias Ried-Larsen, Søren Johannes Sørensen

The following supporting information is available for this article:

**Figure S1**. Genera that contributed the most to the increased richness.

**Figure S2**. Changed beta diversity between treatments over time.

**Figure S3**. Changes in group dispersion/variance (mean weighted UniFrac distance to group centroid in multivariate space) compared to baseline.

**Figure S4**. The individual variation and individual development based on weighted UniFrac distance.

**Figure S5**. Changes in clinical characteristics over time.

**Figure S6**. Changes in GLMED score during the trial.

**Figure S7**. The alpha diversity across different GLMED scores.

**Figure S8**. The differences in gut microbiota composition between GLMED score decrease and increase groups over time.

**Figure S9**. Trial profile showing the allocation of participants between treatments over time.

**Figure S10**. Rarefaction curves for observed richness and Shannon diversity.

**Figure S11.** The microbial composition of the mock community (a) and the DNA extraction and PCR negative controls (b).

**Table S1**. Changes in clinical characteristics for participants allocated to lifestyle intervention and standard care from baseline to 12-month follow-up.

**Table S2**. The glucose-lowering medications and corresponding scores.

**Table S3.** Genera that significantly changed their relative abundances at M3 or M12 for each treatment.

**Table S4**. The correlations between the changes in clinical characteristics and the changes in gut microbiota alpha diversity.

**Table S5**. Changes in taxa abundance at time windows between GLMED score decrease and increase groups.

**Table S6**. Baseline clinical characteristics of participants allocated to lifestyle intervention and standard care.

**Supplementary figures and tables**


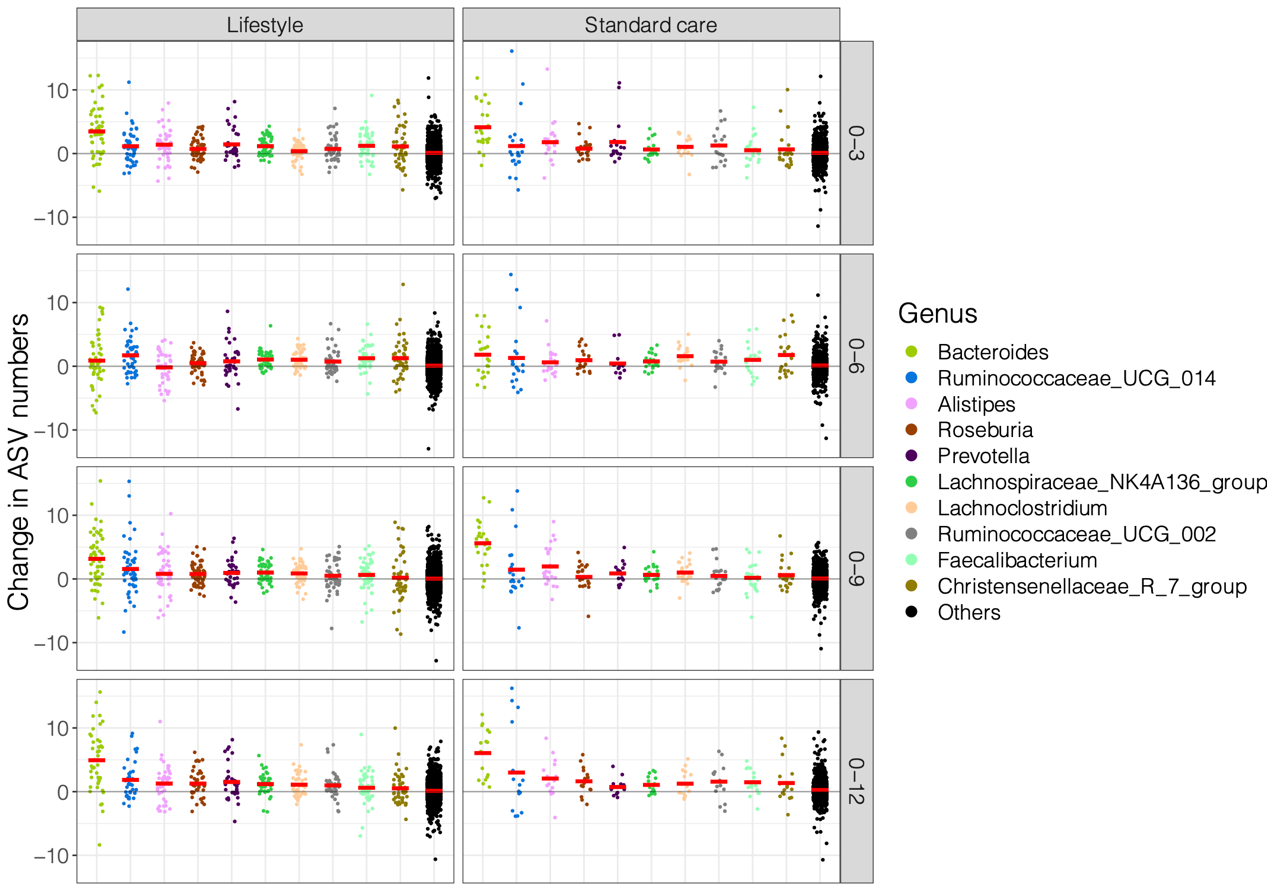


**Figure S1. Genera that contributed the most to the increased richness.** Y axis is the change in ASV numbers for patients within time windows (rarefied at ASV level, colored/grouped at genus level). X axis is the top 10 genera having the highest mean value for changes in ASV numbers. Each dot is a patient with the corresponding ASV number change in a genus. The red bar is the mean value of changes in a genus. Genera having low value for change in ASV numbers were merged as “Others”.


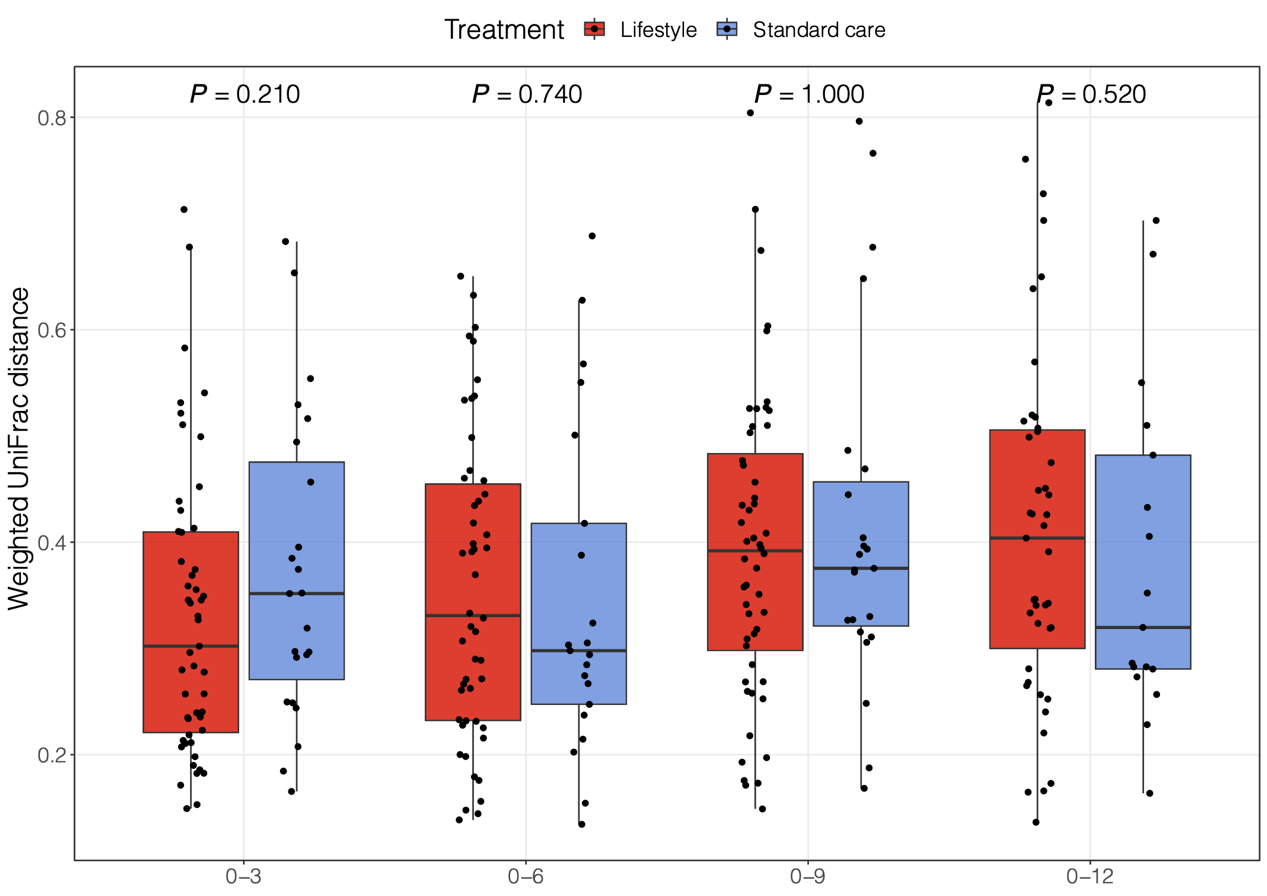


**Figure S2. Changed beta diversity between treatments over time.** Each dot is a weighted UniFrac distance for a patient between two time points and grouped/colored based on the treatment. The comparison of changes in distances between treatments were tested with the Wilcoxon rank sum test.

**Figure S3. Changes in group dispersion/variance (mean weighted UniFrac distance to group centroid in multivariate space) compared to baseline.** The dots refer to the mean and error bars refer to the 95% confidence interval. For the sake of interpretation, if the error bar overlaps the horizontal line (value of zero), it indicates that the change in group dispersion is not significantly different from zero (no change). The comparison of group dispersion between a time point and the baseline for a group was tested with paired two-sided t-test. The comparison of change in group dispersion between groups was performed with two-sided t-test and the significance was shown as “ns” (*P* > 0.05).

**
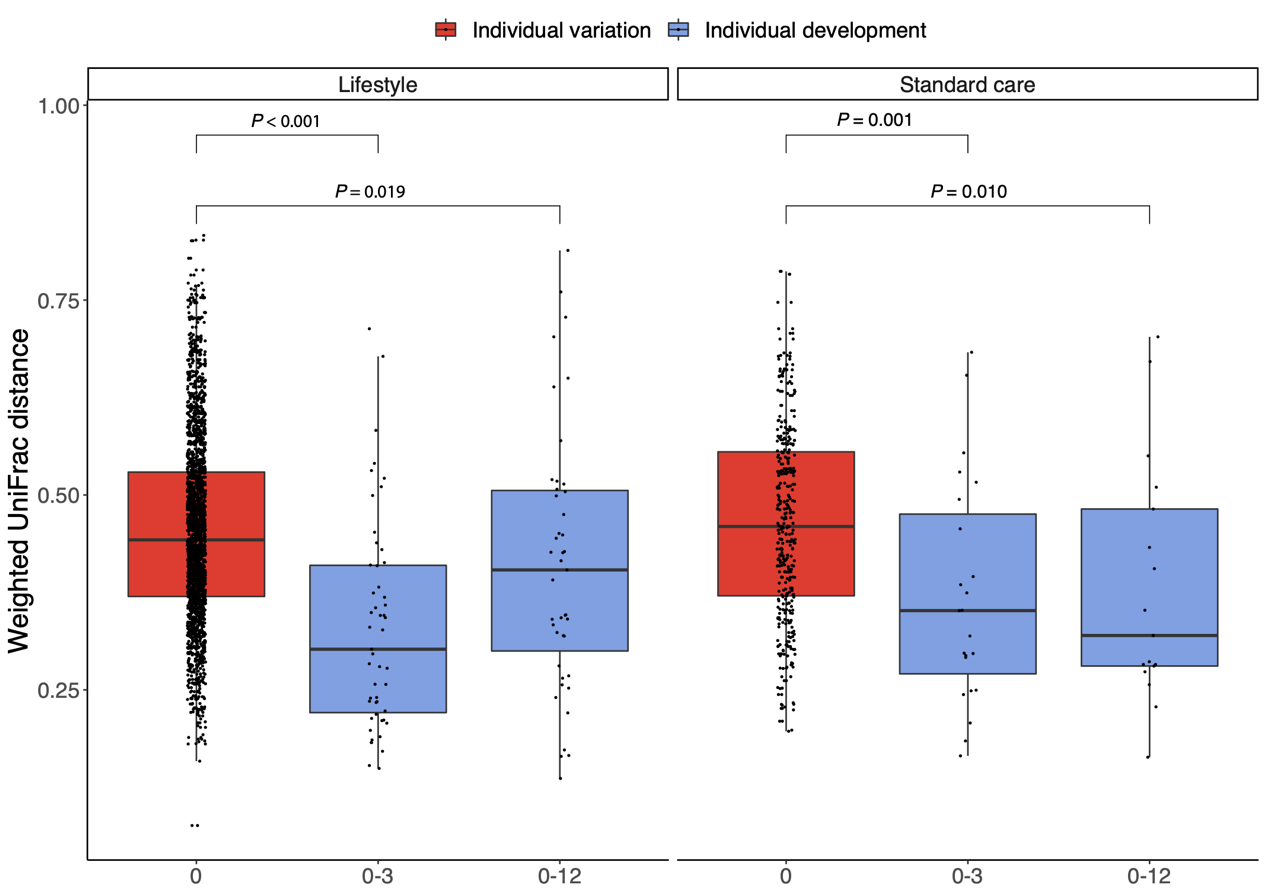
**

**Figure S4. The individual variation and individual development based on weighted UniFrac distance.** The individual variation at baseline M0 was determined based on the weighted UniFrac distance between individuals (inter-individual variation) in a group (lifestyle or standard care). The individual development was determined as the weighted UniFrac distance within each individual but between time points (intra-individual development). Each dot refers to a pairwise weighted UniFrac distance. The comparisons of distances were tested with Wilcoxon rank sum test.

**Figure S5. Changes in clinical characteristics over time.** Colors refer to different treatments. The dots refer to the mean change in clinical characteristics compared to M0 and error bars refer to the 95% confidence interval of mean. For the sake of interpretation, if the error bar overlaps the horizontal line (value of zero), it indicates that the change in clinical characteristics is not significantly different from zero (no change). The comparison of clinical characteristics between a time point and the baseline for a group was tested with paired two-sided t-test. The comparison of change in clinical characteristics between treatments was performed with two-sided t-test and the significance was shown as ns (*P* > 0.05), * (*P* < 0.05), ** (*P* < 0.01), *** (*P* < 0.001). The unit or range of each characteristic is shown in the parentheses. Medication score for glucose, lipid, and pressure ranges from 0 to 7, 0 to 6, and 0 to 8, respectively. A higher score indicates a more intensive pharmacological treatment.

**
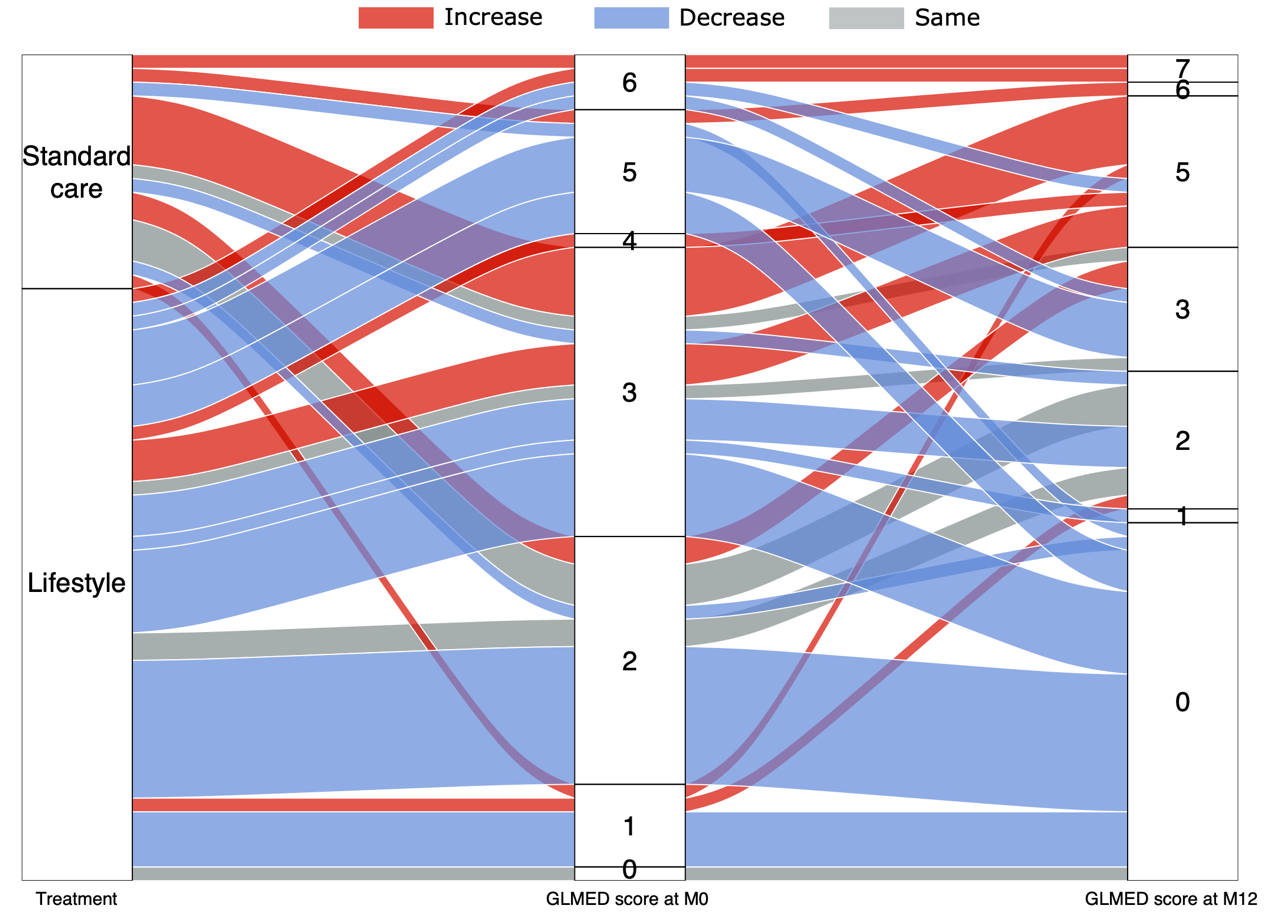
**

**Figure S6. Changes in GLMED score during the trial.** Numbers 0 to 7 are the GLMED score (more details in **Table S2)**. Increase, decrease, and same indicate the different types of change in medication score (M12 *vs.* M0) and colored differently. Standard care and lifestyle are the treatments. Each line/alluvium represents a condition with corresponding values in each column.

**
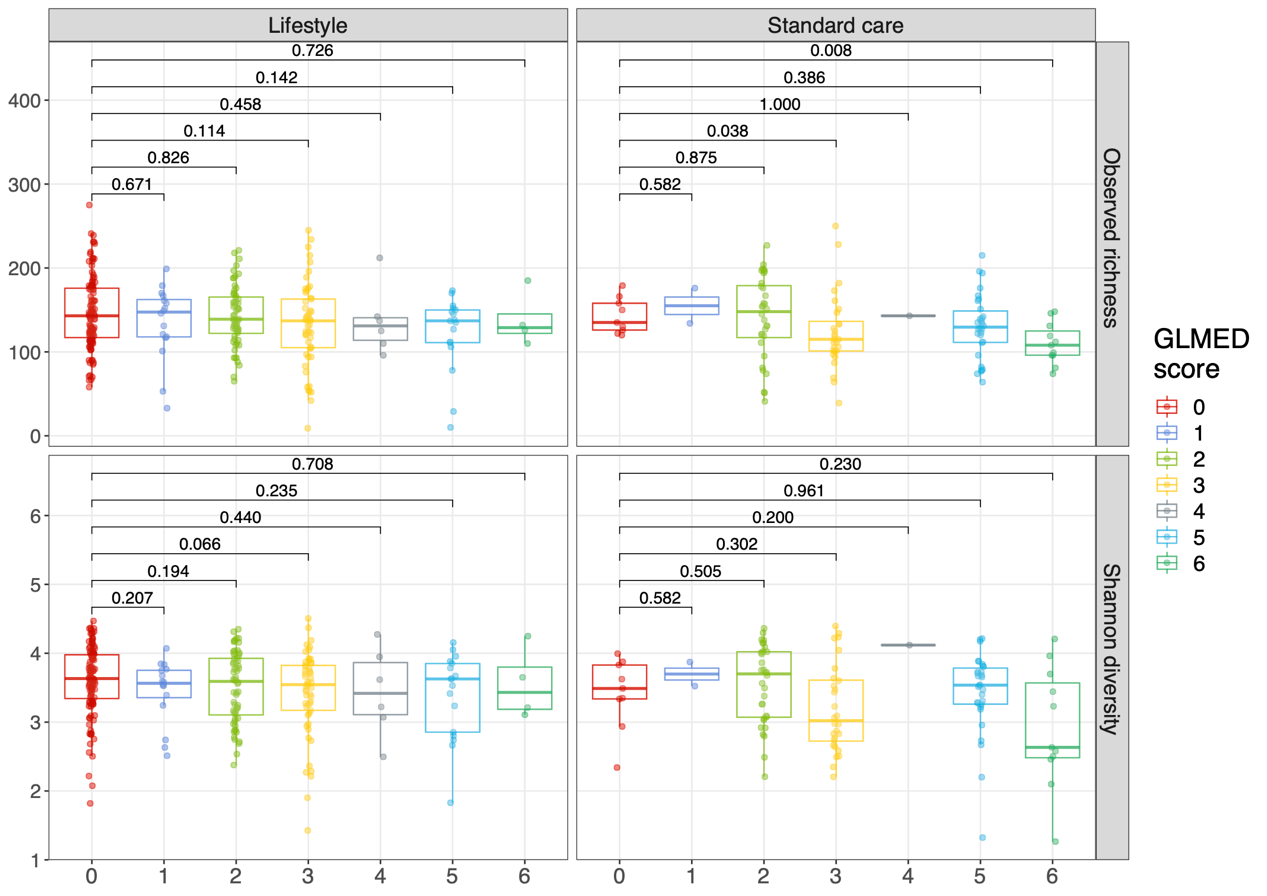
**

**Figure S7. The alpha diversity across different GLMED scores.** The observed richness and Shannon diversity were compared between different medication intensities (GLMED score from 1 to 6) and medication discontinuation (GLMED score of 0). The alpha diversity was shown with box-plot, and the median value of alpha diversity was compared with the paired Wilcoxon rank sum test.

**Figure S8. The differences in gut microbiota composition between GLMED score decrease and increase groups over time.** (a) Changes in alpha diversity for each individual averaged within groups compared to baseline (M0). (b) Changes in group dispersion for each individual averaged within groups compared to baseline. For (a) and (b), the dots refer to the mean and error bars refer to the 95% confidence interval. For the sake of interpretation, if the error bar overlaps the horizontal line (value of zero), it indicates that the change in alpha diversity or group dispersion is not significantly different from zero (no change). The comparison of alpha diversity between a time point and the baseline for a GLMED score group was tested with paired two-sided t-test. The comparison of change in alpha diversity or group dispersion between groups was performed with two-sided t-test and the significance was shown as ns (*P* > 0.05), * (*P* < 0.05), ** (*P* <0.01). (c) Distribution of samples based on weighted UniFrac distance visualized with principal coordinates analysis (PCoA) with ellipses encircling 75% of samples per condition. The *P* value is from the multivariate permutational analysis of variance (PERMANOVA).

**Figure S9. Trial profile showing the allocation of participants between treatments over time.** In total 98 participants were randomly allocated to the intensive lifestyle intervention or the standard care group. Fecal samples and clinical characteristics were collected at baseline (M0), 3- (M3), 6- (M6), 9- (M9), and 12-month (M12) follow-up. Eventually, 86 participants (N = 60 [lifestyle] *vs.* N = 26 [standard care]) who had at least three fecal samples among five time points were included in the analysis.

**Figure S10. Rarefaction curves for observed richness and Shannon diversity.** The observed richness almost reached saturation before 20,000 reads, whereas Shannon diversity was close to maximum after 1000 reads. The black vertical lines show the sequencing depth of 2000 reads. Dots are the mean of alpha diversity in corresponding groups and linked by lines. Error bars are the standard deviation of alpha diversity obtained from 100 random rarefactions at corresponding sequencing depth.

**
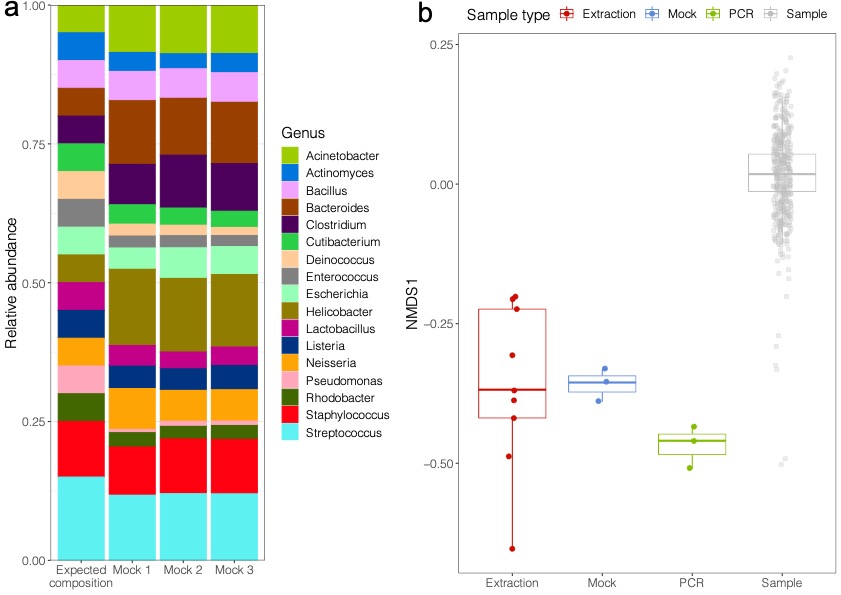
**

**Figure S11.** **The microbial composition of the mock community (a) and the DNA extraction and PCR negative controls (b).** (a) The genomic DNA from Microbial Mock Community B (Even, Low Concentration) v5.1L was used in the sequencing runs. The theoretical composition was named as “Expected composition” and these sequenced mock communities were named as “Mock 1/2/3”. These 20 bacterial strains in each mock were shown at the genus level. (b) Distribution of samples based on Bray-Curtis distance visualized with non-metric multidimensional scaling (NMDS). Sample types are negative DNA extraction controls, negative PCR controls, mock community, and patient samples regardless of treatments and time points.

**Table S1. Changes in clinical characteristics for participants allocated to lifestyle intervention and standard care from baseline to 12-month follow-up.**

|  | **Lifestyle intervention (N = 60)** | **Standard care (N = 26)** | ***P* value** |
| --- | --- | --- | --- |
| **Glycemic control** |  |  |  |
| Hemoglobin A1c (mmol/mol) | -3.4 (7.5) | -2.5 (9.2) | 0.672 |
| fasting glucose (mg/dl) | -15.0 (28.5) | -17.1 (36.3) | 0.801 |
| fasting insulin (uIU/ml) | -5.3 (7.3) | -2.8 (6.5) | 0.13 |
| 2-h glucose (mg/dl) | -17.8 (27.6) | -25.3 (38.8) | 0.387 |
| **Lipids** |  |  |  |
| low-density lipoprotein (LDL) (mg/dl) | 12.3 (31.6) | 10.8 (46.4) | 0.884 |
| high-density lipoprotein (HDL) (mg/dl) | 8.5 (10.2) | 6.7 (11.2) | 0.487 |
| Triglycerides (mg/dl) | 18.3 (62.5) | 13.7 (65.0) | 0.766 |
| **Glucose-lowering medication** |  |  |  |
| Number of individuals with reduction in glucose-lowering medication (n) | 47 (78.3) | 6 (23.1) | **< 0.001** |
| Glucose-lowering medication score | -1.6 (1.6) | 0.5 (1.9) | **< 0.001** |
| **Lipid-lowering medication** |  |  |  |
| Number of individuals with reduction in lipid-lowering medication (n) | 23 (38.3) | 10 (38.5) | 1.000 |
| Lipid-lowering medication score | -0.1 (2.0) | 0.0 (2.0) | 0.709 |
| **Physical fitness** |  |  |  |
| Maximal oxygen consumption (ml/kg/min) | 6.5 (5.7) | 0.3 (4.9) | **< 0.001** |
| **Body composition** |  |  |  |
| BMI (kg/m^2^) | -2.0 (2.4) | -0.82 (2.3) | **0.035** |
| body mass (kg) | -6.2 (7.3) | -2.4 (6.8) | **0.027** |
| fat mass (kg) | -6.3 (6.5) | -1.5 (4.7) | **< 0.001** |
| lean body mass (kg) | 0.7 (2.4) | -1.1 (2.7) | **0.005** |
| gynoid fat mass (kg) | -0.9 (1.0) | -0.2 (0.6) | **< 0.001** |
| android fat mass (kg) | -0.8 (0.9) | -0.2 (0.7) | **< 0.001** |
| fat percentage (%) | -4.9 (4.7) | -0.7 (2.5) | **< 0.001** |
| fat free mass (kg) | 0.7 (2.3) | -0.8 (2.4) | **0.015** |

Data are presented as mean (standard deviation) or n (percentage). Abbreviation: BMI, body mass index (calculated as body mass/weight in kilograms divided by height in meters squared). Medication score ranges: glucose-lowering medication (GLMED), 0 to 7; lipid-lowering medication, 0 to 6. A higher medication score indicates a more intensive pharmacological treatment. *P* values were obtained with t-test (for continuous values) or Chi-square test (for categorical and proportional values). *P* values are in bold if smaller than 0.05.

**Table S2. The glucose-lowering medications and corresponding scores.**

| **Score** | **Medications** |
| --- | --- |
| 7 | 2000 mg metformin + 1.8 mg Victoza + insulin |
| 6 | 2000 mg metformin + 1.8 mg Victoza |
| 5 | 2000 mg metformin + 1.2 mg Victoza |
| 4 | 2000 mg metformin + 0.6 mg Victoza |
| 3 | 2000 mg metformin |
| 2 | 1000 mg metformin |
| 1 | 500 mg metformin |
| 0 | Pause |

**Table S3. Genera that significantly changed their relative abundances at M3 or M12 for each treatment.**

| **Taxa** | **Treatment** | **M0-M3** | | **M0-M12** | |
| --- | --- | --- | --- | --- | --- |
|  |  | **Median difference** | ***P_adj_*** | **Median  difference** | ***P_adj_*** |
| Eubacterium_ eligens_group | Lifestyle | 0.0023 | 0.002 | 0.0017 | 0.015 |
| Eubacterium_ eligens_group | Standard care | 0.0010 | 0.001 | 0.0014 | 0.025 |
| Eubacterium_ ruminantium_group | Lifestyle | NA | NA | 0.0079 | 0.036 |
| Eubacterium_ xylanophilum_group | Lifestyle | 0.0014 | 0.001 | NA | NA |
| Acinetobacter | Lifestyle | NA | NA | 0.0012 | 0.023 |
| Actinomyces | Standard care | NA | NA | -0.0008 | 0.038 |
| Adlercreutzia | Lifestyle | NA | NA | -0.0005 | 0.021 |
| Adlercreutzia | Standard care | -0.0007 | < 0.001 | -0.0006 | 0.012 |
| Agathobacter | Lifestyle | NA | NA | 0.0165 | 0.045 |
| Alistipes | Lifestyle | NA | NA | 0.0161 | 0.026 |
| Alistipes | Standard care | 0.0045 | 0.005 | 0.0111 | 0.038 |
| Bacteroides | Lifestyle | 0.0201 | 0.020 | 0.0445 | < 0.001 |
| Bacteroides | Standard care | 0.0238 | 0.024 | NA | NA |
| Barnesiella | Lifestyle | 0.0031 | 0.003 | 0.0058 | 0.005 |
| Barnesiella | Standard care | 0.0025 | 0.002 | 0.0027 | 0.017 |
| Bifidobacterium | Lifestyle | -0.0218 | < 0.001 | -0.0252 | < 0.001 |
| Bifidobacterium | Standard care | -0.0354 | < 0.001 | NA | NA |
| Blautia | Lifestyle | -0.0308 | < 0.001 | NA | NA |
| Butyricimonas | Standard care | 0.0014 | 0.001 | 0.0008 | 0.025 |
| Clostridiales_family_ XIII_UCG_001 | Lifestyle | 0.0008 | < 0.001 | 0.0012 | < 0.001 |
| Clostridiales_family_ XIII_UCG_001 | Standard care | NA | NA | 0.0006 | 0.021 |
| Collinsella | Lifestyle | -0.0023 | < 0.001 | -0.0033 | 0.002 |
| Coprococcus | Lifestyle | 0.0032 | 0.003 | 0.0047 | 0.010 |
| Corynebacterium | Lifestyle | -0.0032 | < 0.001 | NA | NA |
| Cutibacterium | Lifestyle | -0.0008 | < 0.001 | -0.0008 | 0.007 |
| Cutibacterium | Standard care | -0.0012 | < 0.001 | NA | NA |
| Dorea | Lifestyle | -0.0032 | < 0.001 | NA | NA |
| Eggerthella | Standard care | -0.0009 | < 0.001 | NA | NA |
| Enterococcus | Lifestyle | NA | NA | -0.0010 | 0.020 |
| Escherichia/Shigella | Lifestyle | NA | NA | -0.0040 | 0.010 |
| Faecalibacterium | Lifestyle | 0.1141 | 0.114 | NA | NA |
| Family_ Lachnospiraceae | Lifestyle | 0.0025 | 0.003 | NA | NA |
| Family_ Ruminococcaceae | Lifestyle | 0.0010 | < 0.001 | NA | NA |
| Flavonifractor | Standard care | 0.0020 | 0.002 | NA | NA |
| Intestinimonas | Standard care | 0.0024 | 0.002 | NA | NA |
| Lachnoclostridium | Lifestyle | 0.0016 | 0.002 | 0.0036 | 0.037 |
| Lachnoclostridium | Standard care | 0.0062 | 0.006 | NA | NA |
| Lachnospira | Lifestyle | 0.0009 | < 0.001 | 0.0020 | < 0.001 |
| Lachnospira | Standard care | NA | NA | 0.0015 | 0.045 |
| Lachnospiraceae_ CAG_56 | Lifestyle | 0.0030 | 0.003 | 0.0038 | 0.002 |
| Lachnospiraceae_ FCS020_group | Lifestyle | NA | NA | 0.0006 | 0.020 |
| Lachnospiraceae_ ND3007_group | Lifestyle | 0.0020 | 0.002 | 0.0018 | 0.002 |
| Lachnospiraceae_ ND3007_group | Standard care | NA | NA | 0.0023 | 0.005 |
| Lachnospiraceae_ NK4A136_group | Lifestyle | 0.0057 | 0.006 | 0.0059 | < 0.001 |
| Lachnospiraceae_ NK4A136_group | Standard care | 0.0058 | 0.006 | 0.0047 | 0.015 |
| Lachnospiraceae_ UCG_001 | Lifestyle | NA | NA | 0.0012 | 0.017 |
| Lachnospiraceae_ UCG_004 | Lifestyle | 0.0011 | 0.001 | NA | NA |
| Lawsonella | Lifestyle | -0.0007 | < 0.001 | -0.0006 | 0.025 |
| Odoribacter | Lifestyle | 0.0003 | < 0.001 | 0.0006 | < 0.001 |
| Order_Bacteroidales | Lifestyle | NA | NA | 0.0025 | 0.038 |
| Order_Mollicutes_ RF39 | Lifestyle | NA | NA | 0.0042 | 0.025 |
| Oscillibacter | Lifestyle | 0.0012 | 0.001 | NA | NA |
| Parabacteroides | Lifestyle | NA | NA | 0.0027 | 0.038 |
| Parabacteroides | Standard care | 0.0011 | 0.001 | 0.0040 | 0.040 |
| Paraprevotella | Lifestyle | 0.0011 | 0.001 | 0.0028 | 0.017 |
| Parasutterella | Lifestyle | 0.0010 | < 0.001 | 0.0009 | 0.047 |
| Parasutterella | Standard care | NA | NA | 0.0017 | 0.038 |
| Phascolarctobacterium | Lifestyle | 0.0030 | 0.003 | 0.0099 | 0.001 |
| Prevotella | Lifestyle | NA | NA | 0.0263 | 0.037 |
| Roseburia | Lifestyle | 0.0082 | 0.008 | 0.0135 | 0.005 |
| Roseburia | Standard care | NA | NA | 0.0243 | 0.015 |
| Ruminiclostridium | Lifestyle | NA | NA | 0.0024 | 0.023 |
| Ruminiclostridium | Standard care | NA | NA | 0.0019 | 0.023 |
| Ruminococcaceae_ UCG_003 | Lifestyle | NA | NA | 0.0012 | 0.021 |
| Ruminococcaceae_ UCG_003 | Standard care | 0.0017 | 0.002 | 0.0015 | 0.016 |
| Ruminococcaceae_ UCG_005 | Lifestyle | 0.0020 | 0.002 | 0.0013 | 0.005 |
| Ruminococcaceae_ UCG_005 | Standard care | NA | NA | 0.0012 | 0.034 |
| Senegalimassilia | Lifestyle | -0.0005 | < 0.001 | -0.0008 | 0.005 |
| Staphylococcus | Lifestyle | NA | NA | 0.0008 | 0.027 |
| Sutterella | Lifestyle | 0.0008 | < 0.001 | 0.0014 | 0.002 |
| Sutterella | Standard care | NA | NA | 0.0008 | 0.025 |
| Terrisporobacter | Lifestyle | NA | NA | 0.0023 | 0.038 |

Changes in the median relative abundances of genera between time points are shown. “NA” (not available) indicates that the genus was not present at the time point, so that the *P* value is also not available. The comparison of relative abundance was tested with the paired Wilcoxon rank sum test and the *P* values were adjusted *via* Benjamini-Hochberg correction (*P_adj_*).

**Table S4. The correlations between the changes in clinical characteristics and the changes in gut microbiota alpha diversity.**

|  | **Lifestyle** | | **Standard care** | |
| --- | --- | --- | --- | --- |
| **Clinical characteristics** | **Observed richness** | **Shannon  diversity** | **Observed richness** | **Shannon  diversity** |
| Android.fat.mass | -6.311 (0.146) | -0.145 (**0.044**) | -0.638 (0.940) | -0.023 (0.878) |
| BMI | -2.087 (0.184) | -0.029 (0.267) | 0.181 (0.953) | 0.008 (0.888) |
| Body.mass | -0.718 (0.172) | -0.010 (0.240) | 0.091 (0.932) | 0.002 (0.894) |
| Fasting.glucose | -3.956 (0.272) | -0.110 (0.069) | 1.738 (0.770) | -0.026 (0.779) |
| Fat.free.mass | 0.769 (0.589) | 0.029 (0.205) | -0.725 (0.756) | -0.040 (0.341) |
| Fat.mass | -0.892 (0.118) | -0.016 (0.084) | 0.232 (0.872) | 0.006 (0.793) |
| Fat.percentage | -1.350 (0.064) | -0.023 (0.054) | 0.212 (0.923) | 0.024 (0.522) |
| GLMED.score | -0.779 (0.698) | 0.009 (0.781) | -0.812 (0.766) | 0.042 (0.382) |
| Gynoid.fat.mass | -4.997 (0.168) | -0.086 (0.150) | 2.523 (0.750) | 0.052 (0.707) |
| HbA1c | -0.062 (0.875) | -0.004 (0.546) | 0.126 (0.809) | -0.003 (0.762) |
| Lean.mass | 0.783 (0.578) | 0.029 (0.203) | -0.702 (0.760) | -0.040 (0.334) |
| Medication.lipid.score | -1.945 (0.201) | -0.019 (0.434) | 0.066 (0.980) | -0.058 (0.214) |
| Medication.pressure.score | -0.845 (0.592) | -0.005 (0.870) | -2.701 (0.620) | -0.095 (0.334) |
| Physical.fitness | 2.504 (0.054) | 0.033 (0.147) | -0.003 (0.999) | 0.005 (0.850) |

Data are presented as coefficients (*P* values, before multiple testing correction). Abbreviation: GLMED, glucose-lowering medication; BMI, body mass index (calculated as weight in kilograms divided by height in meters squared); HbA1c: hemoglobin A1c. Only one *P* value is smaller than 0.05 (in bold). The time points were grouped into time windows, namely M0-M3, M0-M6, M0-9, and M0-M12. The correlations between characteristics and alpha diversity were tested *via* linear mixed models, the effects of age, gender, and time windows were adjusted.

**Table S5. Changes in taxa abundance at time windows between GLMED score decrease and increase groups.**

| **Time** | **Taxa** | **Taxonomic level** | **GLMED decrease** | **GLMED increase** | ***P_adj_*** |
| --- | --- | --- | --- | --- | --- |
| 0-3 | Firmicutes | Phylum | 0.0552 | -0.0895 | 0.046 |
| 0-3 | Peptostreptococcaceae | Family | -0.0002 | -0.0080 | 0.009 |
| 0-3 | Clostridiaceae | Family | 0.0003 | -0.0029 | 0.045 |
| 0-6 | Peptostreptococcaceae | Family | 0.0023 | -0.0065 | 0.005 |
| 0-6 | Clostridiaceae | Family | 0.0014 | -0.0023 | 0.039 |
| 0-9 | Clostridiaceae | Family | 0.0029 | -0.0030 | < 0.001 |
| 0-9 | Clostridium | Genus | 0.0029 | -0.0025 | 0.002 |
| 0-12 | Peptostreptococcaceae | Family | 0.0041 | -0.0050 | 0.005 |

Participants from intention-to-treat groups (intensive lifestyle intervention and standard care) were re-grouped based on the change in GLMED score (decrease and increase). The median change in relative taxa abundances at all assessed taxonomic levels (from phylum to ASV) were compared with the Wilcoxon rank sum test, and only the significant taxa are shown here. The *P* values were adjusted *via* the Benjamini-Hochberg correction (*P_adj_*).

**Table S6. Baseline clinical characteristics of participants allocated to lifestyle intervention and standard care.**

|  | **Lifestyle intervention (N = 60)** | **Standard care (N = 26)** | ***P* value** |
| --- | --- | --- | --- |
| **Age** | 53.2 (9.1) | 57.4 (8.1) | **0.037** |
| **Male** | 32 (53.3) | 13 (50.0) | 0.976 |
| **Glycemic control** |  |  |  |
| Hemoglobin A1c (mmol/mol) | 48.8 (9.0) | 50.4 (10.0) | 0.482 |
| fasting glucose (mg/dl) | 137.2 (30.6) | 146.7 (34.2) | 0.235 |
| fasting insulin (ulU/ml) | 18.1 (7.9) | 18.8 (8.5) | 0.739 |
| 2-h glucose (mg/dl) | 144.6 (34.1) | 162.31 (43.0) | 0.075 |
| **Lipids** |  |  |  |
| low-density lipoprotein (LDL) (mg/dl) | 94.2 (30.1) | 85.6 (34.8) | 0.294 |
| high-density lipoprotein (HDL) (mg/dl) | 47.2 (13.5) | 49.7 (15.0) | 0.484 |
| Triglycerides (mg/dl) | 134.0 (60.1) | 143.1 (92.7) | 0.658 |
| **Glucose-lowering medication (n)** |  |  |  |
| None | 1 | 0 |  |
| Biguanide | 48 (80.0) | 21 (80.8) | 1.000 |
| Biguanide and GLP-1 analogue | 11 (18.3) | 5 (19.2) | 1.000 |
| Biguanide, GLP-1 analogue, and insulin | 0 | 0 |  |
| **Glucose-lowering medication score** | 2.9 (1.3) | 3.0 (1.3) | 0.766 |
| **Lipid-lowering medication (n)** |  |  |  |
| None | 12 (20.0) | 4 (15.4) | 0.938 |
| Statin | 48 (80.0) | 22 (84.6) | 0.971 |
| **Lipid-lowering medication score** | 3.0 (2.0) | 3.2 (1.8) | 0.641 |
| **Physical fitness** |  |  |  |
| Maximal oxygen consumption (ml/kg/min) | 28.8 (6.8) | 26.9 (6.8) | 0.265 |
| **Body composition** |  |  |  |
| BMI (kg/m^2^) | 54.9 (7.1) | 55.6 (7.8) | 0.710 |
| body mass (kg) | 31.6 (3.9) | 32.2 (4.5) | 0.547 |
| fat mass (kg) | 35.7 (9.0) | 36.3 (11.3) | 0.830 |
| lean body mass (kg) | 55.9 (10.3) | 56.0 (9.4) | 0.992 |
| gynoid fat mass (kg) | 5.4 (1.5) | 5.4 (2.1) | 0.925 |
| android fat mass (kg) | 4.0 (1.1) | 4.2 (1.3) | 0.453 |
| fat percentage (%) | 38.9 (7.7) | 38.8 (9.0) | 0.991 |
| fat free mass (kg) | 59.2 (10.7) | 59.2 (9.9) | 0.994 |

Data are presented as mean (standard deviation) or n (percentage). Abbreviation: BMI, body mass index (calculated as body mass/weight in kilograms divided by height in meters squared). Medication score ranges: glucose-lowering medication (GLMED), 0 to 7; lipid-lowering medication, 0 to 6. A higher medication score indicates a more intensive pharmacological treatment. *P* values are obtained with t-test (for continuous values) or Chi-square test (for categorical and proportional values). *P* values are in bold if smaller than 0.05.
